# Economic and Humanistic Burden of Multimorbidity in the United States: A Longitudinal Study of Expenditure and Quality of Life Trajectories, 2019–2022

**DOI:** 10.3390/ijerph22121870

**Published:** 2025-12-16

**Authors:** Ibrahim Alliu, Subash Thapa, Lili Yu, Blerta Shehaj, Olamide Asifat

**Affiliations:** 1 Department of Biostatistics, Epidemiology, and Environmental Health Sciences, Jiann-Ping Hsu College of Public Health, Georgia Southern University, Statesboro, GA 30460, USA; st13974@georgiasouthern.edu (S.T.); lyu@georgiasouthern.edu (L.Y.); oa02624@georgiasouthern.edu (O.A.); 2 Department of Health Policy & Community Health, Jiann-Ping Hsu College of Public Health, Georgia Southern University, Statesboro, GA 30460, USA; bs07726@georgiasouthern.edu

**Keywords:** multimorbidity, health-related quality of life, healthcare expenditures

## Abstract

**Highlights:**

**Public health relevance—How does this work relate to a public health issue?**

- Multimorbidity is an increasing public health challenge associated with rising health care utilization and declining quality of life.
- This longitudinal study leverages nationally representative data (2019–2022) to determine how specific combinations of discordant diseases (e.g., cancer co-occurring with respiratory disease) drive divergent trajectories in healthcare expenditures and quality of life.

**Public health significance—Why is this work of significance to public health?**

- This study provides longitudinal evidence of persistent disparities in healthcare expenditures and quality of life among multimorbid individuals.
- Findings identify population groups experiencing disproportionately high costs and poorer health-related quality of life.

**Public health implications—What are the key implications or messages for practitioners, policy makers and/or researchers in public health?**

- Health systems and payers should adopt cluster-based risk segmentation to target high-volatility profiles (such as the cancer + respiratory cluster) for proactive case management and admission prevention.
- Findings support the need for integrated, patient-centered approaches to managing chronic disease in adults.

**Abstract:**

This study examines the economic and humanistic burden associated with multimorbidity among adults in the United States. Using data from the 2019–2022 Medical Expenditure Panel Survey (MEPS), we identified individuals with two or more chronic conditions and assessed trends in healthcare expenditures, out-of-pocket costs, inpatient stays, and health-related quality of life (HRQL). Weighted analyses were conducted to estimate national patterns and annual changes across survey years. Outcomes were analyzed using generalized estimating equation (GEE) models with AR(1) working correlation to compare adjusted mean total and out-of-pocket expenditures, inpatient utilization, and mental and physical HRQL across multimorbidity profiles while controlling for sociodemographic and health factors. Findings showed that multimorbidity was associated with substantial economic burden, reflected in higher healthcare costs and out-of-pocket spending over time. HRQL consistently declined throughout the study years, highlighting the growing humanistic toll of chronic disease clustering. These findings provide longitudinal evidence of persistent disparities associated with multimorbidity and may inform future research and health system planning strategies. The results provide timely evidence for health policymakers and practitioners seeking to improve health system efficiency and equity in managing multimorbidity.

## 1. Introduction

Multimorbidity (MM), defined as the coexistence of two or more chronic conditions, represents a growing public health concern in the United States. According to recent data from the Centers for Disease Control and Prevention (CDC), almost six in every ten American adults have at least one chronic disease, and more than half of these report multiple chronic conditions, accounting for most of the national healthcare expenditures and healthcare utilization [1,2]. As the prevalence of MM rises, healthcare systems face increasing pressure to manage complex care needs while containing costs and preserving quality of life. The costs are not only additive, but the comorbidity of diseases makes clinical management difficult, resulting in increased utilization of health resources, higher expenses, and a greatly reduced quality of life. As the incidence of MM accelerates, especially among young adults, the pattern of national spending on healthcare is widely recognized to be unsustainable, and there is a need for better and better-focused management approaches [3].

While chronic disease has historically been viewed as a condition of aging, recent evidence demonstrates a substantial and sustained rise among younger adults. From 2013 to 2023, multiple chronic conditions (MCC) prevalence among adults aged 18 to 34 rose notably, from 21.8% to 27.1%. The increasing pattern, largely due to rising rates of obesity and depression, is a concerning change in the national health picture [2]. The increasing incidence is compounded by inherent clinical problems of treating MM. The presence of MCC complicates clinical management, increases the risk of polypharmacy and adverse drug events, and leads to conflicting and/or time-intensive treatment regimens that can also impact patients’ treatment adherence [4,5].

While conventional clinical endpoints have objective parameters, such as morbidity and mortality, a full assessment of MM requires measuring the humanistic burden, which quantifies the subjective patient experience throughout the course of disease, and the treatment intervention, on the other hand, becomes more pertinent to the evaluation of care. These can be predominantly evaluated by Patient-Reported Outcomes, such as the Health-Related Quality of Life (HRQL), a multidimensional construct that assesses an individual’s perceived physical and mental functioning, which are central variables of the current study. Alongside this humanistic toll is the direct financial burden placed on patients. This is usually measured by out-of-pocket (OOP) costs, a payment highly concentrated among individuals with more serious conditions and lower health status, so that individuals with the largest need for services tend to have the lowest ability to pay for them [6,7].

Despite the expanding literature on MM, much of the existing evidence remains cross-sectional, limiting insight into how the burden of disease evolves over time. Static estimation hides heterogeneity across disease profiles and fails to capture within-person changes in economic and quality-of-life outcomes. As a result, policymakers and health systems lack longitudinal evidence needed to design risk-stratified, value-based care strategies that target populations with the highest resource needs.

Furthermore, most studies rely on disease counts as a proxy for clinical complexity. While useful, this approach may overlook meaningful variation in burden associated with specific combinations of conditions. Emerging evidence demonstrates that disease patterns differ substantially in terms of mortality risk, functional decline, and healthcare utilization, suggesting that MM configuration may be as important as disease quantity in predicting outcomes. For instance, cancer represents a distinct dimension of MM due to its high treatment intensity, survivorship complexity, and long-term financial impact. Comorbidity involving cancer has been associated with reduced access to curative treatment and worse clinical outcomes, particularly when combined with cardiometabolic or respiratory disease [8]. Accordingly, cancer-related MM warrants separate evaluation within population-based health system analyses.

MM is not inevitable, and multinational cohort evidence demonstrates that adherence to healthy lifestyle behaviors substantially reduces the risk of developing combined cardiometabolic disease, cancer, and diabetes [9]. These findings highlight the role of prevention alongside healthcare delivery reforms.

The present study addresses these gaps by examining longitudinal trajectories of healthcare expenditures, out-of-pocket spending, inpatient utilization, and HRQL across defined MM clusters in a nationally representative U.S. cohort. Using four years of data from the MEPS, this study compares burden across disease profiles rather than simple disease counts, with the aim of informing risk-targeted clinical management and policy strategies.

## 2. Materials and Methods

### 2.1. Study Design and Data Source

This study utilized a retrospective, longitudinal cohort design using the 2019–2022 MEPS Panel 24 longitudinal data file. MEPS is a nationally representative survey of the U.S. civilian noninstitutionalized population, administered by the Agency for Healthcare Research and Quality (AHRQ), which collects detailed person-level information on health status, healthcare utilization, and expenditures.

Panel 24 follows individuals for four consecutive years, allowing the evaluation of within-person changes in economic, humanistic, and utilization outcomes. Survey design features, including clustering, stratification, and longitudinal person-level weights, were incorporated in all analyses to produce nationally representative estimates.

### 2.2. Study Population and MM Clusters

The study population included all 5565 individuals in the Panel 24 cohort. At baseline (Year 1: 2019), individuals were categorized into mutually exclusive MM clusters based on the presence of chronic conditions within three clinical domains.

#### 2.2.1. Disease Ascertainment

Chronic conditions were identified using the MEPS self-reported physician-diagnosed conditions. MEPS captures disease status through standardized variables that indicate whether a respondent was ever told by a doctor or healthcare professional that they had a specific condition. This approach is standard in disease surveillance.

Conditions were conceptually grouped using the Clinical Classification Software Refined (CCSR) framework, which maps ICD-10-CM diagnosis codes into clinically meaningful disease categories. In MEPS, these conditions are operationalized as “priority conditions” based on self-report rather than raw ICD coding. Therefore, while classification is conceptually derived from ICD-10 via CCSR groupings, disease status in this study was determined using survey-based diagnosis indicators. A chronic condition was considered present if the corresponding MEPS variable was coded as “yes” in any survey wave.

#### 2.2.2. Disease Domains

Chronic illnesses were grouped into three clinical domains:

1. Cardiometabolic disease was defined using four MEPS diagnosis indicators: Diabetes mellitus, coronary heart disease, hypertension, and hyperlipidemia. A respondent was classified as having cardiometabolic disease if at least one indicator was equal to 1.
2. Respiratory diseases included asthma and emphysema.
3. Cancer was defined using both the general cancer diagnosis variable and site-specific cancer indicators, including bladder, breast, lung, prostate, colorectal, and lymphatic malignancies.

#### 2.2.3. Disease Timeframe and Treatment Status

MEPS disease variables reflect lifetime diagnosis rather than current disease activity. Consequently, the cancer domain includes respondents with active disease, individuals currently receiving treatment, and cancer survivors in remission.

##### MM Cluster Construction

Three binary disease indicators (cardiometabolic, respiratory, and cancer) were constructed and used to define mutually exclusive multimorbidity (MM) clusters based on domain membership. Initially, seven MM profiles were specified:

1. No target conditions;
2. Respiratory only;
3. Cardiometabolic only;
4. Cancer only;
5. Cardio–respiratory;
6. Cancer–respiratory combination;
7. Cancer–cardiometabolic and three-domain multimorbidity (cancer + cardiometabolic + respiratory).

To ensure adequate sample size and model stability, individuals in the cancer–cardiometabolic and all three conditions combined groups were merged into a single high-complexity cancer combination category (“Rare Cancer Combination”). This aggregation reduced sparse cell counts and mitigated quasi-separation in regression models while preserving clinical interpretability.

#### 2.2.4. Disease Burden vs. Disease Count

Recognizing that clustering based on disease groupings may mask heterogeneity in disease severity or multiplicity within categories (e.g., having both diabetes and heart disease while being classified as “cardiometabolic only”), we conducted sensitivity analyses incorporating within-domain disease counts for cardiometabolic, respiratory, and cancer conditions. These analyses allowed us to distinguish effects attributable to disease combinations from effects driven simply by the number of conditions present.

### 2.3. Outcome Variables

Five primary outcomes were analyzed to provide a comprehensive assessment of the MM burden as follows:

- Economic burden: Measured by (I) Total Annual Healthcare Expenditures (sum of all the payments from all sources) and (II) Total Annual OOP spending (direct payments made by patients/families). All costs were inflation-adjusted to 2022 U.S. dollars using the Medical Care component of the Consumer Price Index (CPI);
- Humanistic burden: Assessed using the Veterans RAND-12 (VR-12) health survey, a validated instrument yielding two summary scores: (I) the Physical Component Summary (PCS) and (II) the Mental Component Summary (MCS). These scores are standardized to a mean of 50, with lower scores indicating poorer quality of life;
- Healthcare utilization: Measured by the annual number of inpatient-hospital stays per person.

### 2.4. Covariates

To control for confounding, all models were adjusted for sociodemographic factors: age, sex, and race/ethnicity. Furthermore, to address the impact of socioeconomic determinants of health, the models included covariates: health insurance status, income status, and educational level.

### 2.5. Statistical Analysis

All statistical analyses were performed using SAS software, Version 9.4, and were weighted using MEPS longitudinal weights to produce nationally representative estimates. To examine the longitudinal trajectories of the five outcomes, a set of Generalized Estimating Equation (GEE) models with an Autoregressive (AR(1)) structure was utilized, which adjusts for the correlation of repeated measures within the same subject over time. All models included the MM cluster, year, and their interaction (Cluster and Year) as primary predictors, while controlling for sociodemographic and socioeconomic factors. Specific statistical distributions (Gamma, Normal, and Negative Binomial) were chosen for each outcome based on its data characteristics. A gamma distribution with a log link was selected for the expenditure models (Total Expenditure and OOP Spending). HRQL scores were modeled using a normal distribution. The number of inpatient stays was modeled using a Negative Binomial distribution to account for the over-dispersed nature of the count data.

#### Sensitivity Analysis

To validate whether observed burdens were driven by specific disease combinations (clusters) or simply the cumulative count of conditions, a sensitivity analysis was conducted. This model included the MM cluster variable while simultaneously adjusting for three categorical indicators: cardiometabolic condition count category, respiratory condition count category, and cancer site count category. These adjustments allowed separation of disease-pattern effects from disease-count effects. Since cancer status was used directly to construct the MM clusters, perfect collinearity between cancer count and cluster assignment was anticipated.

### 2.6. Missing Data Handling

MEPS uses negative values to denote non-substantive responses, including −1: Inapplicable; −2: Not ascertained; −7: Refused; −8: Don’t know; and −15: Cannot be computed. These values were treated as missing. expenditure, insurance, and income variables were used as provided in the MEPS public-use files, which incorporate AHRQ’s internal imputation procedures for item nonresponse. HRQL scores were analyzed using complete-case observations only.

## 3. Results

### 3.1. Baseline Characteristics of the Study Population

The study cohort included 5565 individuals representing a weighted national population of 336.9 million U.S. civilians for the 2019–2022 period. The baseline demographic and clinical characteristics of the population, stratified by the defined MM clusters, are detailed in Table 1. The mean age of the population at baseline (2019) was 38.26 years (95% CI, 37.11–39.40). Almost three-fifths of the population (59.76%) was classified as having none of the targeted chronic conditions (Cluster 0). The most prevalent single condition group was cardiometabolic disease only (Cluster 2), representing 25.08% of the weighted population. The Rare Cancer Combination group (Cluster 6) represented 1.12% of the population. Socioeconomic factors varied significantly by cluster; for example, insurance coverage and income status showed distinct distributions across the MM profiles.

### 3.2. System-Level Economic Burden: Total Healthcare Expenditures

After adjusting for socioeconomic and demographic covariates, the model showed significant differences in inflation-adjusted total healthcare expenditures across MM clusters. The Type 3 score test indicated a highly significant main effect of cluster (χ^2^ = 62.54, *p* < 0.001). The main effect for time (year) was not statistically significant, and the cluster-year interaction was also non-significant, indicating that relative differences between clusters remained stable over time rather than diverging or converging across the 2019–2022 period. 

Age was a statistically significant covariate (χ^2^ = 55.73, *p* < 0.001), whereas race/ethnicity, sex, insurance status, education level, and poverty category were not statistically significant in the fully adjusted model (Table 2).

**Figure 1:**
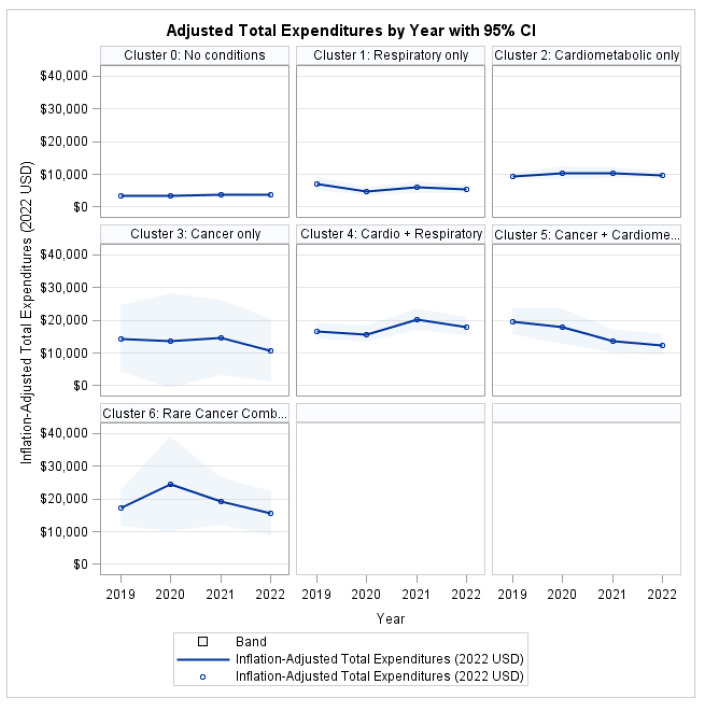
Total Health Expenditures by Profile and Year.

As shown in Figure 1, individuals without the target conditions consistently exhibited the lowest expenditure throughout the follow-up period, whereas all MM clusters incurred higher costs. The Rare Cancer Combination group (Cluster 6) demonstrated the greatest economic burden, with mean expenditures consistently exceeding those of the single-condition clusters across all study years. The 95% confidence intervals illustrate a clear separation between the low- and high-burden groups, while also reflecting greater variability in the high-complexity category.

### 3.3. Patient Financial Burden: OOP Expenditures

After adjustment for demographic and socioeconomic covariates, no statistically significant differences in out-of-pocket expenditures were observed across MM clusters (χ^2^ = 9.74, *p* = 0.1361; Table 3). Likewise, the Cluster × Year interaction was non-significant (χ^2^ = 23.57, *p* = 0.1696), indicating that trajectories of out-of-pocket spending remained parallel across groups over time.

In contrast, insurance status exerted a statistically significant protective effect (χ^2^ = 5.57, *p* = 0.0182), confirming its critical role in shielding patients from healthcare-related financial exposure. Income status, educational attainment, age, sex, and race/ethnicity also demonstrated significant associations with out-of-pocket burden (all *p* < 0.05), underscoring the predominance of socioeconomic determinants over disease-based classifications.

**Figure 2:**
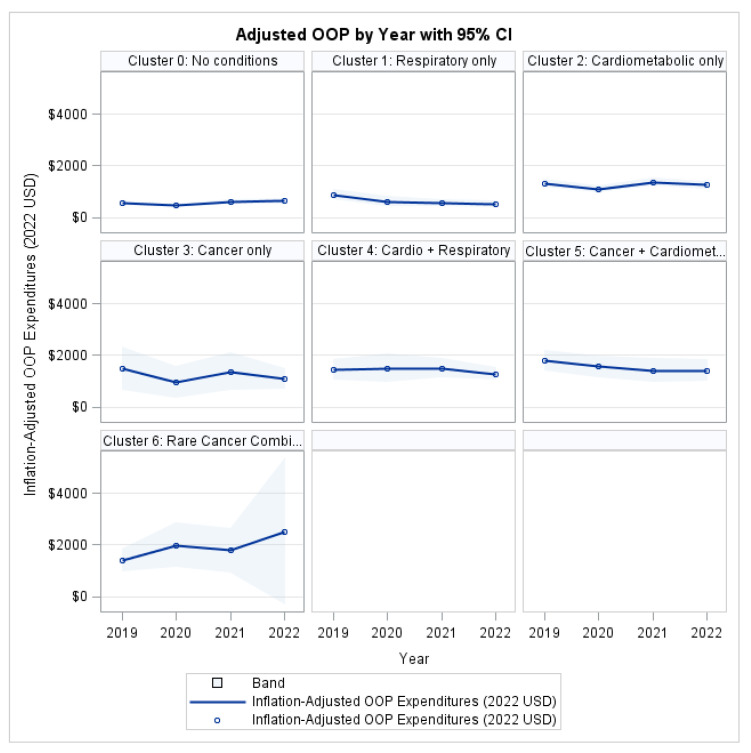
Out-of-Pocket Expenditures by Profile and Year.

As illustrated in Figure 2, although individuals with MM consistently exhibited higher absolute OOP costs than healthy peers, the time trends remained largely parallel across all clusters, supporting the absence of a significant cluster-specific trajectory effect.

### 3.4. Humanistic Burden: Physical HRQL

To quantify the physical health impact of MM, the analysis demonstrated a highly significant main effect of MM on PCS (χ^2^ = 93.82, *p* < 0.001), indicating substantial heterogeneity in physical health status across disease profiles. In contrast, the overall effect of time was not statistically significant (χ^2^ = 3.05, *p* = 0.3837; Table 4), suggesting no uniform improvement or deterioration in physical health across the population during the 2019–2022 period.

The cluster-year interaction approached but did not reach conventional statistical significance (χ^2^ = 28.51, *p* = 0.0547). This result indicates no strong statistical evidence that physical health trajectories differed systematically by cluster over time, although borderline significance suggests possible heterogeneity in slopes.

Several covariates were significantly associated with PCS. Age exhibited a strong inverse association with physical health (χ^2^ = 142.50, *p* < 0.001). Significant differences were also observed by race/ethnicity (χ^2^ = 13.76, *p* = 0.0081) and sex (χ^2^ = 7.55, *p* = 0.0060). Socioeconomic variables demonstrated robust associations with physical health outcomes, with both education level (χ^2^ = 83.01, *p* < 0.001) and poverty category (χ^2^ = 30.97, *p* < 0.001) emerging as significant predictors. In contrast, insurance status was not significantly associated with PCS after adjustment (χ^2^ = 0.73, *p* = 0.3940).

**Figure 3:**
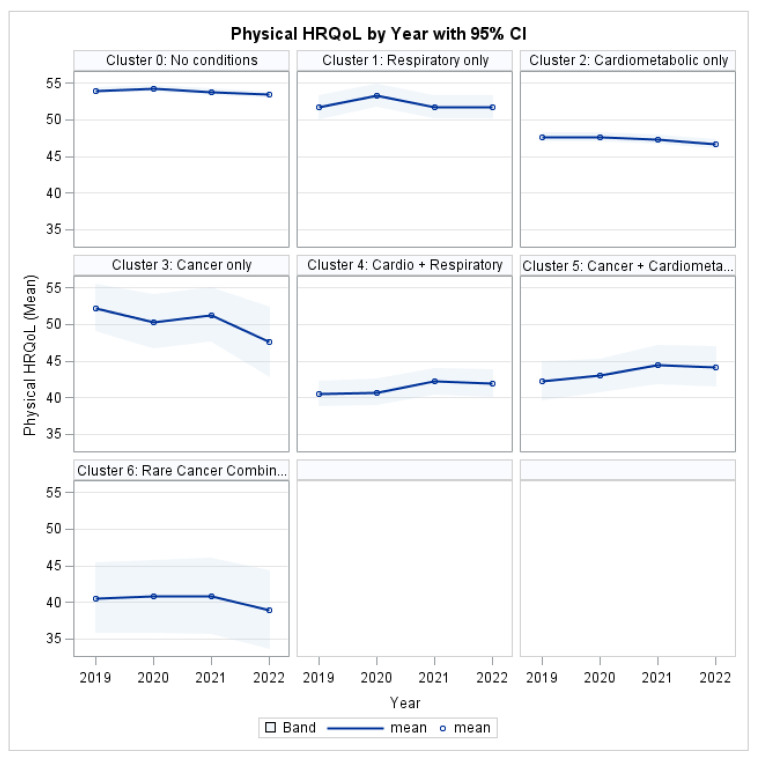
Physical HRQL by Profile and Year.

As illustrated in Figure 3, individuals with MM consistently reported lower PCS scores than the reference group (Cluster 0: No target conditions) throughout the study period. The most pronounced physical impairment was observed among individuals in the Cardio-Respiratory cluster and the Rare Cancer Combination group, with deficits persisting across all four years.

### 3.5. Humanistic Burden: Mental HRQL

The Type 3 analysis revealed a statistically significant main effect of MM Cluster on MCS (χ^2^ = 63.14, *p* < 0.001; Table 5), indicating substantial differences in mental health burden across disease profiles. In contrast, there was no significant overall effect of time (χ^2^ = 3.02, *p* = 0.3880), demonstrating that mental health scores did not change uniformly across the cohort between 2019 and 2022.

The cluster-year interaction was not significant (χ^2^ = 19.12, *p* = 0.3842), indicating no statistical evidence that mental health trajectories differed by cluster over time. This means there are persistent between-group differences in MCS levels rather than differential worsening or improvement across clusters.

Several individual-level characteristics were independently associated with mental health outcomes. Age was significantly associated with MCS (χ^2^ = 31.37, *p* < 0.001), with older individuals reporting lower mental health scores. Significant differences were also observed across race/ethnicity (χ^2^ = 31.19, *p* < 0.001) and sex (χ^2^ = 17.61, *p* < 0.001). Socioeconomic status was a strong determinant of mental health, with both education level (χ^2^ = 16.31, *p* = 0.0225) and poverty category (χ^2^ = 27.84, *p* < 0.001) reaching statistical significance. In contrast, insurance status was not significantly associated with MCS in the fully adjusted model (χ^2^ = 0.37, *p* = 0.5407).

**Figure 4:**
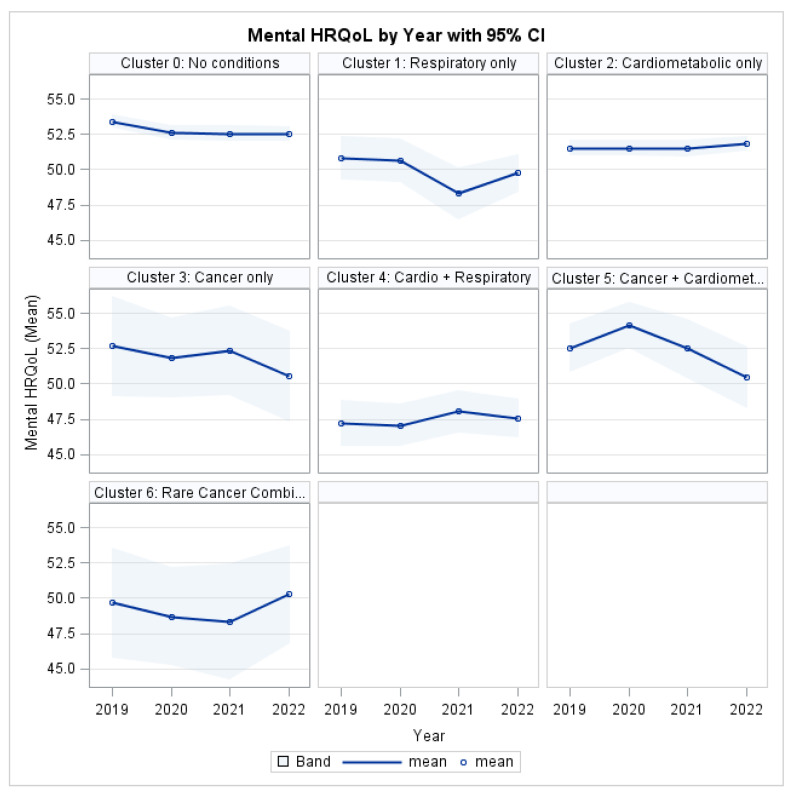
Mental HRQL by Profile and Year.

As shown in Figure 4, MM clusters consistently exhibited lower MCS scores relative to the reference group throughout follow-up. The greatest mental health deficits were observed among individuals in the Cardio-Respiratory and Rare Cancer Combination clusters, with impairments persisting across all four years.

### 3.6. Healthcare Utilization: Inpatient Stays

To examine healthcare utilization associated with MM, generalized estimating equation models were used to assess the longitudinal burden of inpatient hospitalizations.

The Type 3 analysis showed a statistically significant main effect of MM Cluster on inpatient utilization (χ^2^ = 45.34, *p* < 0.001; Table 6), indicating substantial differences in hospitalization burden across disease profiles. There was no significant overall time effect (χ^2^ = 2.67, *p* = 0.4450), suggesting that hospitalization rates did not change uniformly between 2019 and 2022.

The Cluster × Year interaction was not significant (χ^2^ = 16.91, *p* = 0.5294), indicating that clusters maintained parallel hospitalization trajectories over time rather than displaying divergent trends. This finding implies that observed differences reflect stable disparities in healthcare utilization across MM groups rather than accelerating or decelerating patterns.

Several patient-level factors were independently associated with hospitalization risk. Age was a significant predictor (χ^2^ = 26.53, *p* < 0.001), with older individuals more likely to experience inpatient admissions. Differences across race/ethnicity were statistically significant (χ^2^ = 18.88, *p* < 0.001), as were differences by sex (χ^2^ = 9.11, *p* = 0.0025). Insurance status was also a significant determinant of inpatient utilization (χ^2^ = 10.98, *p* < 0.001), reflecting differential access to hospital-based care.

Socioeconomic gradients were evident in hospitalization patterns: poverty category showed a strong association with inpatient utilization (χ^2^ = 36.23, *p* < 0.001). Education level exhibited borderline significance (χ^2^ = 12.49, *p* = 0.0856), suggesting a potential but weaker relationship with hospitalization risk in the fully adjusted model.

**Figure 5:**
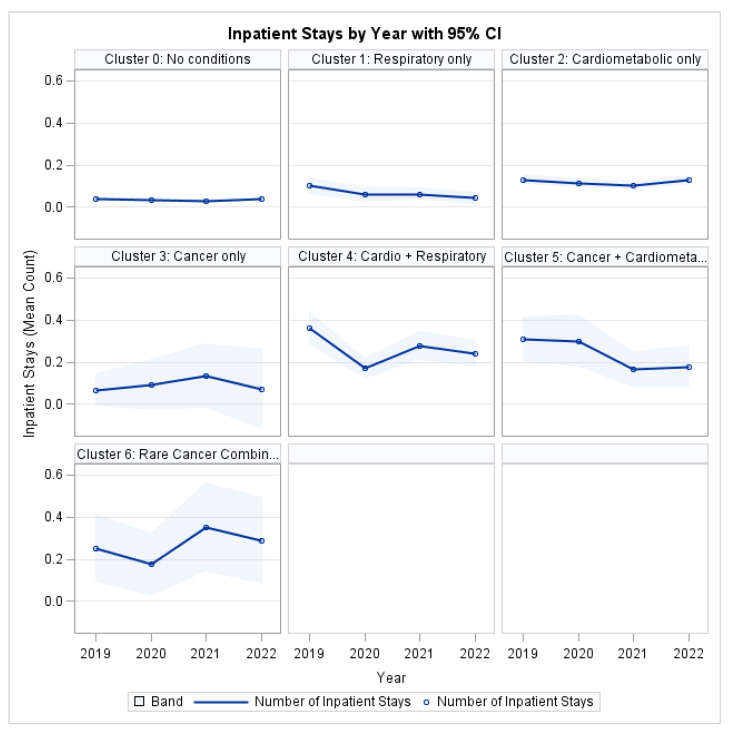
Inpatient Stays by Profile and Year.

As illustrated in Figure 5, all MM clusters demonstrated higher inpatient utilization compared to the reference group (Cluster 0: No target conditions). The highest hospitalization burden was observed in the Cardio-Respiratory and Rare Cancer Combination clusters, which consistently exceeded single-condition groups across the study period.

### 3.7. Multivariable Model Estimates

Table 7 presents the parameter estimates for the five multivariable models. Across outcomes, calendar year indicators showed no consistent associations with total expenditures, out-of-pocket spending, or inpatient utilization. Small declines were observed in mental and physical HRQL in later years.

MM profiles were strongly associated with total expenditures and quality-of-life outcomes. Clusters involving cardiometabolic and respiratory conditions, as well as cancer-related combinations, showed the largest increases in expenditures and the greatest reductions in both physical and mental HRQL. Out-of-pocket spending showed weaker associations, suggesting that patient financial burden was influenced more by insurance and socioeconomic factors rather than by disease pattern.

Interaction terms between clusters and survey years were generally not significant, indicating that outcome differences across disease profiles reflected persistent level differences rather than diverging trajectories. Covariate associations followed expected patterns: older age, lower income, and lower educational attainment were linked to higher utilization and reduced HRQL.

### 3.8. Sensitivity Analyses: Role of Disease Counts Within Axes

To assess whether observed associations were driven by specific MM patterns or simply by the number of chronic conditions within each disease domain, all GEE models were re-estimated, with additional categorical disease-count variables. Results from the joint score statistics for sensitivity models are presented alongside the primary Type 3 results in Table 2, Table 3, Table 4, Table 5 and Table 6.

In sensitivity analyses including within-domain condition count categories, cardiometabolic and respiratory counts provided additional explanatory information beyond the MM cluster variable. In contrast, the cancer count category was perfectly collinear with cancer-related cluster membership and therefore could not be estimated independently. This reflects the construction of the clustering scheme, in which all individuals with cancer were, by design, assigned to cancer-related profiles.

#### 3.8.1. Total Healthcare Expenditures (Table 2)

For total healthcare expenditures, the association between MM profile and cost burden remained statistically significant after accounting for within-domain disease counts. Specifically, cluster membership remained an independent predictor of total expenditures in the sensitivity model (χ^2^ = 21.11, *p* = 0.0018), indicating that the pattern of co-occurring diseases explained additional variation in expenditures beyond disease burden alone.

In addition, cardiometabolic disease count was independently associated with higher expenditures (χ^2^ = 10.47, *p* = 0.0053), while respiratory count showed a smaller but statistically significant contribution (χ^2^ = 4.87, *p* = 0.0273). These findings suggest that both the configuration of comorbid conditions and disease load within cardiometabolic and respiratory domains contribute meaningfully to health system costs.

#### 3.8.2. Out-of-Pocket Expenditures (Table 3)

For out-of-pocket expenditures, the MM cluster was not a statistically significant predictor in either the primary model (χ^2^ = 9.74, *p* = 0.1361) or the sensitivity model (χ^2^ = 3.51, *p* = 0.7424). Similarly, cardiometabolic and respiratory disease counts were not independently associated with OOP spending (χ^2^ = 5.04, *p* = 0.0803; χ^2^ = 0.03, *p* = 0.8622).

This pattern indicates that patients’ OOP burden is shaped more strongly by socioeconomic and coverage factors than by disease configuration or disease count, consistent with the substantial effects observed for insurance status, education, and poverty category.

#### 3.8.3. Physical HRQL (PCS) (Table 4)

For physical HRQL, the cluster remained a strong independent predictor after accounting for disease counts. Cluster effects remained highly significant in both the primary (χ^2^ = 93.82, *p* < 0.001) and sensitivity models (χ^2^ = 29.62, *p* < 0.001), demonstrating that the organization of conditions across domains has a distinct association with physical functioning.

In addition, both cardiometabolic and respiratory disease counts were independently associated with worse physical health (χ^2^ = 29.54, *p* < 0.001; χ^2^ = 14.44, *p* < 0.001), indicating that cumulative burden within these domains contributes additively to physical decline. These findings suggest that both disease pattern and disease intensity are important determinants of physical HRQL.

#### 3.8.4. Mental HRQL (MCS) (Table 5)

For mental HRQL, the cluster remained statistically significant in the sensitivity analysis, though attenuated relative to the primary model (primary: χ^2^ = 63.14, *p* < 0.001; sensitivity: χ^2^ = 17.62, *p* = 0.0072). Respiratory disease count also demonstrated a strong independent association with mental health (χ^2^ = 8.70, *p* = 0.0032), whereas cardiometabolic disease count was not statistically significant (χ^2^ = 2.16, *p* = 0.3391).

These findings indicate that mental HRQL is influenced by both MM configuration and respiratory disease burden, specifically suggesting that respiratory conditions may exert disproportionate psychological or functional effects compared to cardiometabolic disease.

#### 3.8.5. Inpatient Hospital Stays (Table 6)

For inpatient hospital stays, the cluster effect was significant in the primary model (χ^2^ = 45.34, *p* < 0.001) but became non-significant after adjusting for within-axis disease counts in the (χ^2^ = 9.27, *p* = 0.1591). This indicates that differences in hospitalization rates across MM profiles are largely explained by disease burden within domains rather than the specific clustering pattern itself. In contrast, cardiometabolic count remained strongly associated with hospitalization risk (χ^2^ = 11.12, *p* = 0.0038), while respiratory disease count was not associated with hospitalization risk (χ^2^ = 0.21, *p* = 0.6480). Cancer burden could not be estimated due to sparse cell counts. This pattern implies that higher hospitalization rates are driven primarily by the *intensity* of cardiometabolic burden rather than by specific MM clusters.

Overall, the sensitivity analyses showed that cluster membership continues to matter the most for total expenditures and HRQL, whereas disease counts, particularly cardiometabolic burden, are more important for hospitalization. OOP spending is largely insensitive to either structure or count of chronic conditions once socioeconomic factors are controlled.

## 4. Discussion

This longitudinal analysis provides nationally representative evidence that MM is associated with substantial and persistent differences in healthcare expenditures, inpatient utilization, and HRQL across clinically defined disease profiles in U.S. adults. While outcome levels differed significantly by MM pattern, trajectories over time remained largely parallel, indicating that inequalities in burden reflect sustained differences across disease groups rather than accelerating divergence during the study period.

### 4.1. Economic Burden and Disease Configuration

MM profile emerged as a central determinant of total healthcare expenditures even after extensive adjustment for demographic and socioeconomic characteristics. Individuals with cardiometabolic, respiratory disease, and cancer-related combinations consistently incurred the greatest costs across all years of follow-up, aligning with prior research demonstrating that such combinations increase treatment complexity, adverse events, and healthcare utilization [5,8]. Importantly, sensitivity analyses demonstrated that disease configurations explained variability in total expenditures beyond disease burden within domains, indicating that pattern-based classification contributes information not captured by disease count alone.

These findings extend evidence from cross-sectional studies that have documented the dominant role of MM in driving healthcare costs nationwide [1,2] and align with international systematic reviews demonstrating an increasing association between healthcare utilization and disease clustering [10]. The disproportionate expenditures observed among cancer-respiratory and cardiometabolic clusters are consistent with clinical evidence indicating that cancer comorbidity limits treatment access and worsens prognosis, particularly in the presence of respiratory or cardiometabolic disease [8]. While mortality was not an outcome in this study, the present cost patterns are congruent with the literature linking these combinations to greater clinical complexity and prolonged healthcare engagement.

Sensitivity analyses further demonstrated that cardiometabolic and respiratory disease burden contributed independently to expenditure variation beyond cluster membership, whereas cancer burden could not be evaluated separately because cluster assignment fully captured cancer status.

### 4.2. Patient-Level Financial Burden

In contrast to total system-level costs, OOP spending did not differ significantly by disease profile after multivariable adjustment. Instead, patients’ financial burden was shaped largely by insurance status, income, education, and demographic characteristics. This pattern mirrors prior findings showing that medical debt and financial hardship are concentrated among uninsured and socioeconomically vulnerable populations rather than being determined solely by disease burden [6,7,11].

Although patients with MM experienced higher absolute personal expenditures, the lack of differential trajectory likely reflects cost-sharing protections, including coverage-based expenditure ceilings. These mechanisms may limit catastrophic financial exposure even as overall system expenditures rise, highlighting a structural trade-off between risk protection for patients and escalating payer burden.

### 4.3. Quality of Life and Functional Burden

Differences in both physical and mental HRQL across MM profiles were pronounced and persistent throughout follow-up. Individuals in cancer-related and cardiometabolic–respiratory clusters exhibited the greatest impairment, reinforcing prior findings that MM is strongly associated with reduced physical functioning, disability, and psychological distress [12,13].

Sensitivity models indicated that disease configuration retained an independent association with HRQL after accounting for within-domain disease count, underscoring that patterns of illness may influence patient experience beyond disease accumulation. Respiratory disease burden exhibited a particularly strong relationship with mental health outcomes, suggesting that symptoms such as dyspnea and activity limitation may exert disproportionate psychosocial effects.

Notably, quality-of-life trajectories were parallel across groups, implying that functional decline is established early in the disease process rather than worsening progressively after MM develops. This interpretation is consistent with prior evidence showing that impairment associated with chronic disease often occurs early and remains stable thereafter [12].

### 4.4. Hospitalization Risk and Disease Intensity

For inpatient utilization, disease burden, rather than configuration, predominated. After adjusting for within-domain disease counts, cluster membership no longer independently predicted hospitalizations, whereas cardiometabolic disease intensity remained significant. This contrasts with findings for expenditures and quality of life and suggests that acute healthcare use may be better explained by disease severity and number rather than disease configuration.

This distinction emphasizes that both burden-based and pattern-based frameworks have relevance for clinical planning. Disease pattern appears informative for long-term economic impact and functional impairment, whereas disease intensity more directly predicts hospital-based utilization.

### 4.5. Implications for Policy, Prevention, and Care Delivery

The results suggest the potential value of disease profile–informed risk stratification and toward disease profile-informed risk stratification strategies. Rather than applying broad strategies to all patients with multiple conditions, identifying individuals with persistently high-burden profiles may allow for more efficient allocation of care coordination and preventive services.

However, current interventions for MM have demonstrated limited effectiveness in improving outcomes or reducing costs. Recent meta-analytic evidence suggests that available management strategies yield, at best, modest benefits, underscoring the need for better-targeted approaches and earlier prevention [14]. Prevention remains a critical complement to treatment-based strategies, as multinational cohort studies demonstrate that healthy lifestyle behaviors substantially reduce the likelihood of developing combined cardiometabolic disease and cancer [9].

Finally, while the present findings are U.S.-based, the implications are likely relevant to other health systems facing rising MM prevalence. Disease pattern recognition may assist systems internationally in designing care pathways that emphasize risk stratification, prevention, and efficiency.

### 4.6. Limitations

This study has several important strengths. It uses nationally representative MEPS longitudinal data, allowing for the evaluation of four-year trajectories in expenditures, utilization, and health-related quality of life. The use of clinically meaningful multimorbidity profiles, standardized HRQL measures, and inflation-adjusted expenditures strengthens comparability across groups and time. Additionally, the simultaneous assessment of economic and humanistic outcomes provides a comprehensive view of the burden of multimorbidity, and the longitudinal design helps distinguish persistent disparities from short-term fluctuations. Finally, sensitivity analyses incorporating within-domain disease burden further enhance the robustness of the findings regarding the independent effect of disease clustering.

However, the study is not without limitations. First, disease ascertainment in MEPS is based on self-reported physician diagnoses rather than clinical records, which may introduce bias or misclassification. However, prior validation studies have demonstrated reasonable agreement between self-report and clinical documentation for major chronic conditions in population-based surveys, and MEPS remains a widely used source for national health surveillance.

Second, the analysis is restricted to the civilian, noninstitutionalized population. Individuals residing in long-term care facilities, nursing homes, or other institutional settings are not captured in MEPS and are likely to have a higher burden of chronic disease and healthcare utilization. Consequently, the estimates presented here may be conservative with respect to the total population burden.

Third, although the models were adjusted for a broad set of sociodemographic and socioeconomic covariates, residual confounding from unmeasured variables remains a possibility. Factors such as disease severity, duration, clinical staging, behavioral risk factors, medication adherence, and health system characteristics were not explicitly available in the dataset and, therefore, could not be incorporated into the models.

Fourth, the MM clusters were constructed using three disease domains, which necessarily simplifies the complex clinical reality. Individuals classified within a single domain may differ meaningfully in terms of disease severity or combinations within that domain (e.g., having both coronary heart disease and diabetes versus a single cardiometabolic condition). Although sensitivity analyses incorporating within-domain disease counts were conducted to address this limitation, classification error and within-group heterogeneity may persist.

Fifth, cancer status in MEPS reflects lifetime diagnosis and does not distinguish between active disease, remission, or treatment phase. Consequently, the cancer-related clusters represent a heterogeneous group that includes survivors as well as individuals undergoing active treatment, which may reduce associations with utilization and quality-of-life outcomes. Also, cancer burden could not be evaluated independently of cluster membership in sensitivity models because all individuals with cancer were assigned to cancer-related clusters by design, resulting in structural collinearity.

Finally, HRQL was measured using the VR-12 instrument, which captures physical and mental functioning but does not provide disease-specific symptom assessment or clinical severity grading. Quality-of-life impairments specific to certain conditions, particularly among oncology or respiratory populations, may therefore be underrepresented.

## 5. Conclusions

This study demonstrates that the MM burden in the United States is patterned and persistent across meaningful disease profiles. While outcome trajectories did not diverge over time, substantial baseline differences in economic burden and quality of life highlight sustained disparities related to disease configuration.

These findings suggest that disease pattern, in addition to disease burden, may inform patient stratification and health system planning. Longitudinal monitoring and prevention-focused strategies remain relevant complements to reform healthcare delivery.

## Figures and Tables

**Table 1:** Baseline Characteristics at Year 1 (2019): Unweighted n (Weighted %) and Weighted N.

Characteristics	Unweighted n (Weighted %)	Weighted N
Clusters		
Cluster 0: No Conditions	2862 (59.76)	201,351,877
Cluster 1: Respiratory Only	384 (7.53)	25,382,546
Cluster 2: Cardiometabolic Only	1723 (25.08)	84,519,951
Cluster 3: Cancer Only	42 (0.58)	1,954,930
Cluster 4: Cardio + Respiratory	368 (4.85)	16,339,563
Cluster 5: Cancer + Cardiometabolic	141 (1.65)	5,566,711
Cluster 6: “Rare Cancer Combination”	87 (1.12)	3,779,979
Total	5565 (100.0)	336,940,627
Gender		
Male	2617 (49.37)	166,358,534
Female	2948 (50.63)	170,582,093
Race/Ethnicity		
Hispanic	1381 (19.2)	64,601,815
Non-Hispanic White	2857 (58.2)	196,242,452
Non-Hispanic Black	884 (12.5)	42,154,385
Non-Hispanic Asian	254 (6.2)	20,970,510
Non-Hispanic Other/Mixed	185 (3.8)	12,971,464
Insurance Status		
Insured	4719 (89.9)	292,270,727
Uninsured	613 (10.1)	32,799,348
Educational Level		
No Degree	756 (9.06)	30,412,716
Less than 16 (Underage)	1046 (24)	80,553,438
GED	181 (2.3)	7,816,499
High School Diploma	1792 (29.7)	99,794,996
Bachelor’s Degree	835 (16.3)	54,856,816
Master’s Degree	412 (8.0)	26,875,052
Doctorate Degree	112 (2.2)	7,394,810
Other Degree Income Status	405 (8.3)	28,013,149
Poor/Negative	1134 (13.5)	44,059,680
Near Poor	318 (4.005)	13,223,863
Low Income	783 (11.7)	38,200,651
Middle Income	1447 (28.12)	91,783,368
High Income	1770 (42.62)	139,109,119

**Table 2:** Type 3 and sensitivity joint tests for inflation-adjusted total healthcare expenditures.

Predictor		Primary		Sensitivity	
	DF	χ^2^	*p*-Value	χ^2^	*p*-Value
Year	3	1.50	0.6817	2.12	0.5489
Cluster	6	62.54	<0.001	21.11	0.0018
Cluster-Year	18	12.89	0.7980	12.31	0.8309
Age	1	55.73	<0.001	43.80	<0.001
Race	4	8.00	0.0914	8.61	0.0717
Sex	1	2.00	0.1577	2.41	0.1207
Insurance Status	1	0.32	0.5697	0.33	0.5662
Educational Level	7	6.17	0.5200	6.99	0.4297
Poverty Cat	4	7.10	0.1308	6.94	0.1392
Cardiometabolic burden (count category)	2	—	—	10.47	0.0053
Respiratory burden (count category)	1	—	—	4.87	0.0273
Cancer burden (count category)	0	—	—	0.00	—

Note: Counts are grouped as 0, 1, or ≥2 except for cancer, which is binary (0 vs. ≥1).

**Table 3:** Type 3 and sensitivity joint tests for out-of-pocket healthcare expenditures.

Predictor		Primary		Sensitivity	
	DF	χ^2^	*p*-Value	χ^2^	*p*-Value
Year	3	1.82	0.6110	10.37	0.0157
Cluster	6	9.74	0.1361	3.51	0.7424
Cluster-Year	18	23.57	0.1696	22.57	0.2075
Age	1	43.97	<0.001	40.18	<0.001
Race	4	29.11	<0.001	28.82	<0.001
Sex	1	5.94	0.0148	6.28	0.0122
Insurance Status	1	5.57	0.0182	5.77	0.0163
Educational Level	7	32.26	<0.001	33.75	<0.001
Poverty Cat	4	36.75	<0.001	38.10	<0.001
Cardiometabolic burden (count category)	2	—	—	5.04	0.0803
Respiratory burden (count category)	1	—	—	0.03	0.8622
Cancer burden (count category)	0	—	—	0.00	—

Note: Counts are grouped as 0, 1, or ≥2 except for cancer, which is binary (0 vs. ≥1).

**Table 4:** Type 3 and sensitivity joint tests for physical HRQL.

Predictor		Primary		Sensitivity	
	DF	χ^2^	*p*-Value	χ^2^	*p*-Value
Year	3	3.05	0.3837	11.56	0.0091
Cluster	6	93.82	<0.001	29.62	<0.001
Cluster-Year	18	28.51	0.0547	28.62	0.0533
Age	1	142.50	<0.001	132.08	<0.001
Race	4	13.76	0.0081	11.57	0.0208
Sex	1	7.55	0.0060	9.10	0.0026
Insurance Status	1	0.73	0.3940	0.42	0.5150
Educational Level	7	83.01	<0.001	73.95	<0.001
Poverty Cat	4	30.97	<0.001	28.81	<0.001
Cardiometabolic burden (count category)	2	—	—	29.54	<0.001
Respiratory burden (count category)	1	—	—	14.44	0.001
Cancer burden (count category)	0	—	—	0.00	—

Note: Counts are grouped as 0, 1, or ≥2 except cancer, which is binary (0 vs. ≥1).

**Table 5:** Type 3 and sensitivity joint tests for mental HRQL.

Predictor		Primary		Sensitivity	
	DF	χ^2^	*p*-Value	χ^2^	*p*-Value
Year	3	3.02	0.3880	6.47	0.0909
Cluster	6	63.14	<0.001	17.62	0.0072
Cluster-Year	18	19.12	0.3842	19.13	0.3836
Age	1	31.37	<0.001	33.57	<0.001
Race	4	31.19	<0.001	29.59	<0.001
Sex	1	17.61	<0.001	18.37	<0.001
Insurance Status	1	0.37	0.5407	0.46	0.4960
Educational Level	7	16.31	0.0225	16.30	0.0225
Poverty Cat	4	27.84	<0.001	26.89	<0.001
Cardiometabolic burden (count category)	2	—	—	2.16	0.3391
Respiratory burden (count category)	1	—	—	8.70	0.0032
Cancer burden (count category)	0	—	—	0.00	—

Note: Counts are grouped as 0, 1, or ≥2 except cancer, which is binary (0 vs. ≥1).

**Table 6:** Type 3 and sensitivity joint tests for inpatient hospital stays.

Predictor		Primary		Sensitivity	
	DF	χ^2^	*p*-Value	χ^2^	*p*-Value
Year	3	2.67	0.4450	1.48	0.6869
Cluster	6	45.34	<0.001	9.27	0.1591
Cluster-Year	18	16.91	0.5294	17.00	0.5231
Age	1	26.53	<0.001	20.87	<0.001
Race	4	18.88	0.001	17.12	0.0018
Sex	1	9.11	0.0025	9.17	0.0025
Insurance Status	1	10.98	<0.001	10.33	0.0013
Educational Level	7	12.49	0.0856	13.88	0.0533
Poverty Cat	4	36.23	<0.001	34.62	<0.001
Cardiometabolic burden (count category)	2	—	—	11.12	0.0038
Respiratory burden (count category)	1	—	—	0.21	0.6480
Cancer burden (count category)	0	—	—	0.00	—

Note: Counts are grouped as 0, 1, or ≥2 except cancer, which is binary (0 vs. ≥1).

**Table 7:** Parameter estimates (β, SE) for multivariable models of expenditures, quality of life, and inpatient utilization.

Predictor	Total Expenditure (β, SE)	OOP Spending (β, SE)	HRQL (β, SE)	MHRQL (β, SE)	Inpatient Stay (β, SE)
Intercept	1.6394 (0.2988) ***	0.1655 (0.2501)	51.841 (0.6679) ***	55.5064 (0.7729) ***	−3.6957 (0.3196) ***
Year 2020	−0.0878 (0.1318)	−0.0951 (0.088)	0.2088 (0.2907)	−0.7259 (0.3271) *	−0.117 (0.1862)
Year 2021	0.0196 (0.1161)	0.1925 (0.1075)	−0.3434 (0.2906)	−0.8623 (0.4135) *	−0.2314 (0.1949)
Year 2022	0.0546 (0.1163)	0.2792 (0.1241) *	−0.8471 (0.3036) **	−0.8893 (0.3922) *	−0.1703 (0.2029)
Year 2019	0	0	0	0	0
Cluster 1	0.6143 (0.3167)	0.4428 (0.194) *	−2.97 (0.9675) **	−2.0464 (1.1865)	0.9 (0.3906) *
Cluster 2	0.2955 (0.1107) **	0.2482 (0.1034) *	−2.6836 (0.5291) ***	−3.0669 (0.5242) ***	0.4252 (0.1877) *
Cluster 3	0.7983 (0.3867) *	0.2535 (0.2905)	0.8423 (1.413)	−2.263 (1.9058)	−0.353 (0.5998)
Cluster 4	0.8477 (0.1356) ***	0.3845 (0.1464) **	−8.882 (1.1141) ***	−7.0427 (1.2378) ***	1.3714 (0.2199) ***
Cluster 5	0.8078 (0.2041) ***	0.2641 (0.1614)	−6.2555 (1.6793) ***	−2.5273 (1.0655) *	1.0251 (0.3412) **
Cluster 6	0.6554 (0.1994) ***	0.1539 (0.1708)	−7.5567 (2.2901) ***	−5.0189 (2.3153) *	0.7784 (0.3882) *
Cluster 0	0	0	0	0	0
Cluster 1 * 2020	−0.3679 (0.3461)	−0.2827 (0.2834)	1.5721 (0.7308) *	−0.5376 (1.0892)	−0.5106 (0.5322)
Cluster 1 * 2021	−0.1865 (0.4016)	−0.5406 (0.2709) *	0.5117 (1.0193)	−1.6289 (1.4962)	−0.3658 (0.436)
Cluster 1 * 2022	−0.1429 (0.356)	−0.6569 (0.2519) **	1.0222 (0.8444)	−0.2179 (1.0701)	−0.7937 (0.5586)
Cluster 1 * 2019	0	0	0	0	0
Cluster 2 * 2020	0.2096 (0.2095)	−0.1083 (0.1205)	−0.2525 (0.438)	0.5686 (0.5058)	−0.0086 (0.248)
Cluster 2 * 2021	0.0284 (0.1555)	−0.1755 (0.1523)	−0.2621 (0.4563)	0.8014 (0.5459)	−0.0062 (0.2426)
Cluster 2 * 2022	0.0203 (0.1434)	−0.3156 (0.1545) *	−0.3481 (0.5148)	1.1999 (0.5388) *	0.1474 (0.2658)
	0	0	0	0	0
Cluster 3 * 2020	−0.4571 (0.4719)	−0.4671 (0.2211) *	−2.9505 (1.3218) *	−0.9183 (1.7336)	0.5186 (0.9603)
Cluster 3 * 2021	−0.2138 (0.4408)	−0.3621 (0.3148)	−2.3027 (1.2667)	0.2669 (1.9602)	1.0066 (0.7945)
Cluster 3 * 2022	−0.5104 (0.4769)	−0.7058 (0.3291) *	−4.0024 (1.8366) *	−0.6806 (1.2431)	0.336 (1.0067)
	0	0	0	0	0
Cluster 4 * 2020	0.0185 (0.179)	0.2184 (0.1801)	−0.3961 (0.6981)	0.3825 (0.8119)	−0.7701 (0.2964) **
Cluster 4 * 2021	0.121 (0.185)	−0.1855 (0.1995)	1.8452 (0.8372) *	1.572 (1.1359)	−0.1425 (0.289)
Cluster 4 * 2022	0.0042 (0.1824)	−0.3973 (0.2114)	1.6648 (1.0005)	1.583 (1.1588)	−0.2905 (0.3004)
	0	0	0	0	0
Cluster 5 * 2020	0.0311 (0.2515)	0.0172 (0.1967)	0.3442 (1.4126)	1.908 (1.0444)	0.1415 (0.3385)
Cluster 5 * 2021	−0.3528 (0.2346)	−0.3801 (0.2385)	2.0813 (1.6573)	0.0674 (1.1054)	−0.495 (0.4914)
Cluster 5 * 2022	−0.3595 (0.2567)	−0.3284 (0.2337)	3.6614 (1.6793) *	−1.4388 (1.1153)	−0.502 (0.4958)
	0	0	0	0	0
Cluster 6 * 2020	0.5145 (0.3369)	0.6363 (0.3253)	−0.1405 (1.3824)	−0.1176 (1.2862)	−0.3143 (0.5769)
Cluster 6 * 2021	0.1524 (0.3142)	0.2486 (0.3151)	0.9395 (2.0668)	0.0705 (1.6144)	0.6911 (0.7746)
Cluster 6 * 2022	−0.0269 (0.2627)	0.399 (0.4359)	−1.1645 (1.9254)	2.1807 (1.6471)	0.446 (0.4983)
Age	0.017 (0.0022) ***	0.0145 (0.0019) ***	−0.1799 (0.0118) ***	0.0695 (0.0125) ***	0.0195 (0.0038) ***
Non-Hispanic White Only	0.1366 (0.1372)	0.3377 (0.0963) ***	−1.1236 (0.4102) **	−2.5622 (0.4848) ***	0.2578 (0.1369)
Non-Hispanic Black Only	−0.0828 (0.1599)	−0.1051 (0.1247)	−1.6349 (0.6772) *	−2.0684 (0.7586) **	0.3037 (0.1788)
Non-Hispanic Asian Only	−0.2312 (0.2084)	0.0162 (0.2105)	−0.3892 (0.6542)	−1.0795 (0.8591)	−0.5849 (0.2774) *
Non-Hispanic other or Multiple race	0.1547 (0.1929)	0.2763 (0.1992)	−3.159 (1.2) **	−4.5762 (1.8109) *	0.6699 (0.3154) *
Hispanic	0	0	0	0	0
Male	−0.1009 (0.0692)	−0.1533 (0.0611) *	0.9432 (0.3435) **	1.574 (0.3735) ***	−0.3 (0.0972) **
Female	0	0	0	0	0
Insured	0.1763 (0.3123)	−0.4573 (0.1626) **	−0.2925 (0.3432)	0.2859 (0.4658)	0.6996 (0.2391) **
Not Insured	0	0	0	0	0
GED	−0.246 (0.203)	−0.3692 (0.1854) *	−0.3603 (1.2369)	−3.0719 (1.4205) *	−0.2891 (0.2715)
High School Diploma	−0.1386 (0.1587)	0.0064 (0.1567)	1.5719 (0.6049) **	−0.0133 (0.7887)	−0.1383 (0.1666)
Bachelor’s Degree	−0.1637 (0.1763)	0.2953 (0.1619)	4.0425 (0.6571) ***	−0.3895 (0.8568)	−0.347 (0.1934)
Master’s Degree	−0.0146 (0.184)	0.3647 (0.1628) *	5.2604 (0.707) ***	0.5715 (0.868)	−0.0448 (0.2121)
Doctorate Degree	−0.0036 (0.2816)	0.2549 (0.2107)	4.7453 (1.0213) ***	1.5504 (1.0668)	−0.3047 (0.337)
Other Degree	0.1966 (0.2605)	0.1664 (0.1772)	2.7293 (0.7824) ***	−0.7796 (0.9432)	−0.2146 (0.2114)
Underage 16	−0.291 (0.2188)	0.1678 (0.1871)	1.9473 (0.9395) *	3.1659 (2.2247)	−0.9439 (0.3275) **
No degree	0	0	0	0	0
Poor	0.16 (0.162)	−0.5473 (0.1026) ***	−2.2308 (0.3912) ***	−2.2475 (0.4524) ***	0.7851 (0.1424) ***
Near Poor	0.009 (0.1068)	−0.5371 (0.1282) ***	−1.1582 (0.4567) *	−1.4672 (0.5017) **	0.7467 (0.1756) ***
Low Income	−0.0791 (0.0998)	−0.2481 (0.0863) **	−1.3477 (0.3755) ***	−1.1105 (0.3749) **	0.7495 (0.1522) ***
Middle Income	−0.1731 (0.0936)	−0.1977 (0.0699) **	−0.5375 (0.2673) *	−1.0593 (0.3021) ***	0.2567 (0.111) *
High Income	0	0	0	0	0

Note: The reference categories are Year = 2019; Cluster = 0; Race/Ethnicity = Hispanic; Sex = Female; Insurance = Uninsured; Education = No degree; Income = High income. * *p* < 0.05, ** *p* < 0.01, *** *p* < 0.001.

## Data Availability

Publicly available datasets were analyzed in this study. These data can be accessed at: https://meps.ahrq.gov/mepsweb/data_stats/download_data_files_detail.jsp?cboPufNumber=HC-245 (accessed on 15 June 2025).

## Abbreviations

CDC	Centers for Disease Control and Prevention
HRQL	Health Related Quality of Life
MCC	Multiple chronic conditions
MEPS	Medical Expenditure Panel Survey
MM	Multimorbidity
OOP	Out of Pocket
GEE	Generalized Estimating Equation
